# Antenatal risk factors for postnatal depression: a prospective study of chinese women at maternal and child health centres

**DOI:** 10.1186/1471-244X-12-22

**Published:** 2012-03-22

**Authors:** Bonnie WM Siu, Shirley SL Leung, Patrick Ip, Se Fong Hung, Michael W O'Hara

**Affiliations:** 1 Department of Psychiatry, Castle Peak Hospital, Hong Kong SAR, China; 2 Department of Health, Hong Kong SAR, China; 3 Community Child Health Unit, Department of Paediatrics and Adolescent Medicine, The University of Hong Kong, Hong Kong SAR, China; 4 Department of Psychiatry, Kwai Chung Hospital, Hong Kong SAR, China; 5 Department of Psychology, The University of Iowa, Iowa City, USA

**Keywords:** Postnatal, Depression, Antenatal, Risk factors, Chinese

## Abstract

**Background:**

Risk factors for postnatal depression (PND) are under-explored in the Chinese populations. There is increasing recognition of the importance of identifying predictive factors during the antenatal period for PND. The present study aimed to identify the risk factors for postnatal depression in a community cohort of Chinese women with special focus on the antenatal risk factors.

**Methods:**

Eight hundred and five Chinese women were interviewed during their third trimester of pregnancy and at around 2 months postnatally. Putative risk factors for PND were collected and the diagnosis of PND was confirmed by the Structured Clinical Interview for DSM-IV Axis I Disorders. The 2-month postnatal depression status was used as the dependent variable for univariate and multivariate analyses against putative risk factors.

**Results:**

Marital dissatisfaction (Relative Risk = 8.27), dissatisfied relationship with mother-in-law (Relative Risk = 3.93), antenatal depressive symptomatology (Relative Risk = 3.90), and anxiety-prone personality (Relative Risk = 2.14) predicted PND in Chinese women independently.

**Conclusions:**

Chinese women tend to keep their own feelings and emotions and it is important to monitor Chinese pregnant women with these predictive risk factors so that PND can be identified early.

## Background

Postnatal depression has substantial impact on the quality of life and social functioning of the mother as well as the emotional and cognitive development of the newborn child [1-3]. Because of the adverse consequences for the mother, the child and the family as a whole, the early identification and management of postnatal depression is an important public health concern. There is increasing recognition of the importance of identifying predictive factors such as psychosocial factors during the antenatal period for postnatal depression [4]. In view of the important impact of psychosocial health on the well-being of pregnant women and the baby, standard assessment tools like the Antenatal Psychosocial Health Assessment form have been widely used in antenatal service in North America [5,6]. Culpepper and Jack (1993) recommended that comprehensive assessment of antenatal psychosocial risk factors should be integrated into routine medical care [7]. Antenatal psychosocial factors that have been found to be associated with postnatal depression include marital discord, current or past emotional, physical, sexual or verbal abuse by partner, lack of social support, pregnancy unwanted by the mother after 20 weeks, stressful life events during pregnancy or near delivery, past or present psychiatric disorder in the mother, antepartum depression, prenatal care not started until the third trimester and poor relationship of the mother with her parents and mother-in-law [8-13]. In the Chinese populations, studies for exploring the antenatal risk factors for postnatal depression are relatively scarce [9,14]. The objective of this study was to identify the risk factors for postnatal depression in a community cohort of Chinese women with special focus on risk factors identifiable during the antenatal period. Identified risk factors should assist healthcare professionals in the early identification of postnatal depression and the prompt delivery of effective interventions at a much earlier stage. Moreover, the results would carry significant public health implications as effective interventions and support programmes aiming at reducing or alleviating the impact of these identifiable risk factors may help to reduce the chance of the subsequent development of postnatal depression.

## Methods

### Recruitment sites

This was a collaborative study between the psychiatric units of Hospital Authority and the Department of Health in Hong Kong. The psychiatric units were those providing the Comprehensive Child Development Service (CCDS) for the early identification and management of postnatal depression in Chinese women in Hong Kong. The study was conducted at the nine Maternal and Child Health Centres (MCHCs) involved in the CCDS [15-19]. MCHCs are the major primary healthcare service sites in the community in Hong Kong provided by the Department of Health for pregnant women and infants and all of them are located in areas nearby to surrounding households. Approval for the conduction of the study was obtained from the Ethics and Research Committee of the Hospital Authority and the Ethics Committee of the Department of Health. The study had been performed in accordance with the ethical standards laid down in the 1964 Declaration of Helsinki.

### Participants

A convenient sample of Chinese women aged between 18 and 50 years attending the nine MCHCs for routine antenatal care at their third trimester during the period 1 August 2009 to 31 August 2010 were assessed for their eligibility to enter the study. Women who were competent to give consent and planned to stay in Hong Kong for at least 6 months after delivery (to ensure that those identified to have postnatal depression in this study could be referred to the appropriate services and receive an adequate period of treatments) were recruited after obtaining their written informed consent.

### Study design

This was a prospective cohort study conducted on a sample of Chinese women receiving an existing antenatal and postnatal service provided by the CCDS in Hong Kong. The participants were assessed twice by medical professionals and were also required to complete self-report questionnaires. The first assessment was conducted by psychiatric nurses at MCHCs with the participants during their routine antenatal care in the third trimester. During the first assessment, sociodemographic data, clinical data, and putative antenatal risk factors for postnatal depression were obtained in the context of a semi-structured interview. The participants also completed the Chinese version of the Edinburgh Postnatal Depression Scale (EPDS) for assessment of antenatal depression. Lee et al. (2004) categorized the third trimester EPDS scores into three levels for assessing the level of antenatal depression: 0-9, nil or insignificant depressive symptoms; 10-14, mild to moderate depressive symptoms; and > 14, severe depressive symptoms and this categorization was adopted in the present study [9,20]. The second interview was conducted at about 2 months after childbirth at MCHCs. The participants completed the Chinese version of the EPDS and were interviewed by psychiatric nurses or psychiatrists. In the second interview, participants were asked about putative perinatal risk factors for postnatal depression that were not asked in the first interview in their third trimester. In addition, the Chinese version of the Structured Clinical Interview for DSM-IV Axis I Disorders (SCID-I) was conducted to determine the presence of postnatal depression [21]. Those diagnosed with postnatal depression by SCID-I would be asked to complete the Chinese version of the Beck Depression Inventory (BDI) for the severity of depression [22].

### Measures

#### Sociodemographic and clinical variables and antenatal risk factors for postnatal depression

A semi-structured interview designed to assess sociodemographic and clinical variables as well as the presence or absence of the putative antenatal risk factors for postnatal depression was developed based on a review of literature on risk factors for PND. Variables included age, place of birth, length of stay in Hong Kong, marital status, educational attainment, employment status, residential area, household size, family income, relationship with parents, marital relationship (relationship with partner, i.e. the father of the baby for those not married), relationship with mother-in-law (relationship with the partner's mother for those not married), stressful life events during pregnancy, medical history, history and treatment of infertility or subfertility, number of previous pregnancies/deliveries/miscarriages/stillbirths and induced abortions, weeks of gestation when prenatal care began, unplanned pregnancy and unwanted pregnancy, antenatal admission, attitude of partner to pregnancy, anxiety-prone personality, history of deliberate self-harm, history of substance misuse, history of mental illness and family history of mental illness. For the assessment of putative antenatal risk factors such as relationship with mother-in-law, Likert measures as adopted in Lee et al. study (2004) were used [9] (e.g. the participant was asked to rate her relationship with mother-in-law as "good", "not good but could cope with it" or "bad and could not cope with it").

#### Edinburgh Postnatal Depression Scale (EPDS)

The Chinese version of the 10-item EPDS was used in this study [20,23]. This version of the EPDS has been validated for use in the Chinese populations and had been used as a screening questionnaire to improve the detection of postnatal depression in the community [20]. In 2004, Lee et al. categorized the third trimester EPDS scores into three levels for assessing the level of antenatal depression and this categorization was adopted in the antenatal assessment in the present study [9]. At around 2 months after childbirth, the participants completed EPDS again. EPDS allows a greater extent of international comparison to be made with other studies on postnatal depression.

#### Structured clinical interview for DSM-IV axis. I disorders (SCID-I)

A semi-structured interview was used to obtain reliable DSM-IV diagnoses [24]. The Chinese version, which has been validated and revealed good reliability between the rater and clinician diagnoses, was used to diagnose major depression in the postnatal period [21].

#### Beck depression inventory (BDI)

A 21-item questionnaire with established psychometric properties used to measure the intensity and severity of depression in patients having psychiatric diagnoses [25]. The validated Chinese version of BDI was adopted in this study as a self-rating questionnaire for the participants to complete [22].

#### Semi-structured interview to assess perinatal risk factors for postnatal depression

Similar to the case for the assessment of antenatal risk factors, a semi-structured interview was developed to assess the presence or absence of putative perinatal risk factors for PND that could not be assessed in the third trimester. Risk variables included mode of delivery, perceived control of the mode of delivery, birth complications, persistent wound pain for more than 2 weeks, low birth weight (< 1.5 kg) of baby, neonatal admission, baby health problems, baby gender, attitude of mother and father towards gender of baby, failed breast-feeding, stress in child care, number of children including the newborn, partner non-participation in baby care, peiyue support practice (i.e. a Chinese custom of being accompanied and assisted by a designated female in the first month post delivery). Likert measures were adopted for the assessment of putative perinatal risk factors such as the attitude of mother towards the gender of the baby (e.g. the mother was asked to rate her attitude towards the gender of baby as "satisfied", "neutral", or "dissatisfied").

#### Statistical analysis

The Statistical Package for the Social Science (SPSS) version 19 was used for data analysis. Descriptive statistics were used to summarize the sociodemographic and clinical characteristics. The variables collected by the semi-structured interviews for putative risk factors were either categorical variables (i.e. presence or absence of the putative risk factor) or numerical variables. The categorical variables and numerical variables and the categorical responses to the Likert measures on these putative risk factors were analysed with PND as the dependent variable (outcome measure). The status of PND at 2-month postpartum was used as the dependent variable for univariate and multivariate analyses against the putative risk factors. For univariate analyses, the risk of PND according to the presence or absence of the risk factors was assessed and the risk ratios were calculated with 95% confidence intervals. For categorical variables, χ^2 ^tests were used for the associations between PND and the individual putative risk factor. For continuous variables, the independent sample t-test was used. For multivariate analyses, factors that yield risk ratios greater than two and with 95% confidence intervals that did not cross one in the univariate analysis were entered into stepwise logistic regression to identify independent risk factors as the best joint model for the prediction of PND. The regression was repeated using backward modelling to test for the stability of the model. The unadjusted odd ratios and confidence intervals for each significant variable in the final model were calculated. The adjusted odds ratios were calculated with the inclusion of all the significant variables in the logistic regression. The level of significance was set at *P *< 0.05 throughout the study.

## Results

### Participants recruited

Of the 1002 antenatal women approached by the investigators, 838 (83.6%) consented to take part in the study and were interviewed antenatally. Altogether 805 were interviewed both antenatally and postnatally with an attrition rate of 3.9%. Amongst those who could not be interviewed at the postnatal period: 13 refused; 17 could not be contacted; 1 had returned to the Mainland China; 1 had intra-uterine death and 1 had neonatal death on day 11. There were no differences between the women who completed and those who did not complete the study on age, marital status, educational attainment, earning status, family income, immigrant status and antenatal depressive symptomatology.

The mean age of the 805 women who completed the study was 30.1 years (s.d. = 4.9) and the mean years of education was 14.6 years (s.d. = 2.6); with 74.3% attained secondary educational level in the Hong Kong schooling system (Table 1). About half (51.4%) of them resided in the relatively rural parts (Yuen Long, Tuen Mun, and Tin Shui Wai districts) of Hong Kong. More than one fifth (21.2%) were new immigrants who had resided in Hong Kong for less than 7 years; all the new immigrants in this study had migrated from the Mainland China. The mean gestational age at delivery was 39.4 weeks (s.d. = 1.2) and the postnatal age at assessment of postnatal depression was 8.8 weeks (s.d. = 2.2). The mean birth weight was 3.2 kg (s.d. = 0.4) and the ratio of male to female babies was 1:1. Most of them (90.3%) had received peiyue support and the majority of them had their mother (39.6%) providing the peiyue support, followed by mother-in-law (25.8%). Table 2 illustrated the EPDS scores according to the categorization by Lee et al. in the third trimester of pregnancy for participants with and without depression. The mean EPDS scores at the antenatal and postnatal assessment were 5.8 (s.d. = 4.6) (range = 0-24) and 5.8 (s.d. = 5.5) (range = 0-28) respectively.

**Table 1:** Sociodemographic characteristics of participants (N = 805)

	n (%)
**Educational level**	
**Primary**	29 (3.6%)
**Secondary**	598 (74.3%)
**Tertiary**	178 (22.1%)
**Marital status**	
**Single**	26 (3.2%)
**Married**	750 (93.2%)
**Cohabited**	24 (3%)
**Divorced**	5 (0.6%)
**Place of residence (district)**	
**Yuen Long**	63 (7.8%)
**Tuen Mun**	188 (23.4%)
**Tin Shui Wai**	163 (20.2%)
**Others**	391 (48.6%)
**Working status**	
**Working**	389 (48.3%)
**Not-working**	416 (51.7%)
**Mode of delivery**	
**Spontaneous**	539 (67%)
**Assisted (vacuum/forceps)**	85 (10.6%)
**Elective Caesarean section**	102 (12.7%)
**Emergency Caesarean section**	79 (9.8%)
**Feeding method**	
**Breastfeeding**	141 (17.5%)
**Formula feeding**	436 (54.2%)
**Mixed feeding (both breastfeeding and formula feeding)**	228 (28.3%)
**Number of children including the newborn baby**	
**One**	465 (57.8%)
**Two**	257 (31.9%)
**Three**	68 (8.4%)
**Four**	9 (1.1%)
**Five**	6 (0.7%)
**Peiyue support**	
**Yes**	728 (90.4%)
**No**	77 (9.6%)

**Table 2:** EDPS scores in the third trimester of pregnancy for participants with and without depression

**EPDS scores according to the categorization by Lee et al**.	Mean EDPS scores (SD) for participants with postnatal depression (n = 126)	Mean EDPS scores (SD) for participants without postnatal depression (n = 679)
**0-9** **(nil or insignificant depressive symptoms)**	5.93 (2.53)	3.77 (2.80)
**10-14** **(mild to moderate depressive symptoms)**	12.08 (1.37)	11.61 (1.42)
**> 14** **(severe depressive symptoms)**	17.78 (2.84)	17.40 (2.13)

*EPDS *Edinburgh Postnatal Depression Scale

*SD *Standard Deviation

### Risk factors for postnatal depression

Among the 805 women who completed the postnatal assessment, 126 (15.7%) were diagnosed to have major depressive episode based on the SCID-I and the mean BDI score for the women with depression was 22.6 (s.d. = 8.4). For univariate analyses, 10 categorical variables (risk factors) were found to be associated with postnatal depression by *χ*^2 ^test (Table 3). Age was the only continuous variable found to be associated with postnatal depression (Table 4). Eleven risk factors (both categorical and continuous variables) including "past depression", "marital dissatisfaction", "absence of peiyue support", "antenatal depressive symptomatology", "dissatisfied relationship with mother-in-law", "antenatal stressful life events", "anxiety-prone personality", "persistent wound pain", "felt stress in child care", "not married", and "younger maternal age" that were found to be associated with postnatal depression in univariate analyses were entered into stepwise logistic regression to explore the independent risk factors for PND. The risk factors "past depression" and "anxiety-prone personality" were found to interact with each other and the effect of "past depression" on the development of PND disappeared when "anxiety-prone personality" was included as one of the independent variables for PND. There were four explanatory variables in the final model (Table 5) and backward stepwise regression analysis yielded the same model. The final model illustrated that postnatal depression was predicted by "marital dissatisfaction", "dissatisfied relationship with mother-in-law", "antenatal depressive symptomatology" and "anxiety-prone personality".

**Table 3:** Risk factors significance by Chi-square

	PND (n = 126)	Non-PND (n = 679)	*χ*^2^	df	RR (95% CI)	p-value
**Past depression**						
**Yes**	26	39	31.75	1	3.59 (2.27-5.68)	< 0.001
**No**	100	640				
**Marital Relationship**						
**Not Good**	35	23	95.54	1	8.27 (5.06-13.50)	< 0.001
**Good**	91	656				
**Peiyue support**						
**No**	22	55	10.76	1	2.16 (1.37-3.40)	0.002
**Yes**	104	624				
**Antenatal depressive symptomatology**						
**Yes**	68	94	106.45	1	3.90 (3.04-4.99)	< 0.001
**No**	58	585				
**Relationship with mother-in-law**						
**Not Good**	67	92	105.28	1	3.93 (3.05-5.04)	< 0.001
**Good**	59	587				
**Antenatal stressful life events**						
**Yes**	39	82	29.65	1	2.56 (1.84-3.57)	< 0.001
**No**	87	597				
**Anxiety-prone personality**						
**Yes**	79	199	52.41	1	2.14 (1.79-2.56)	< 0.001
**No**	47	480				
**Persistent wound pain**						
**Yes**	30	67	19.49	1	2.41 (1.64-3.55)	< 0.001
**No**	96	612				
**Felt stress in child care**						
**Yes**	89	218	66.87	1	2.20 (1.88-2.57)	< 0.001
**No**	37	461				
**Married**						
**No**	16	39	8.08	1	2.21 (1.28-3.83)	0.006
**Yes**	110	640				

*PND *Postnatal Depression

*RR *Relative risk

*CI *Confidence Interval

**Table 4:** Risk factors significance by t-test

	PND (n = 126) Mean (s.d.)	Non-PND (n = 679) Mean (s.d.)	t	df	p-value
**Age**	28.76 (4.85)	30.32 (4.84)	-3.31	803	0.001

*PND *Postnatal Depression

**Table 5:** Risk factors selected by stepwise logistic regression

Risk factor	Unadjusted odds ratio (95% CI)	Adjusted odds ratio (95% CI)	p-value
**Marital dissatisfaction**	11.09 (6.27-19.62)	6.44 (3.42-12.15)	< 0.001
**Dissatisfied relationship with mother-in-law**	7.25 (4.79-10.95)	4.74 (2.97-7.57)	< 0.001
**Antenatal depressive symptomatology**	7.30 (4.83-11.02)	4.09 (2.54-6.59)	< 0.001
**Anxiety-prone** **personality**	4.05 (2.73-6.03)	2.27 (1.42-3.65)	0.001

*CI *Confidence Interval

## Discussion

### Prevalence of postnatal depression and its early detection

Postnatal depression was originally thought to be absent in the Chinese populations because of the traditional practice of "peiyue support (doing the month)" during which additional social support is provided by the family for women in the first month after delivery [26]. In 1998, Lee et al. found that the prevalence of PND in Chinese women six weeks after delivery was 5.5% which was relatively low as compared with studies of PND in non-Chinese women [20,27-29]. Lee et al. (1998) elaborated that the low prevalence rate could be related to the protective effect of peiyue support preventing the occurrence of PND in early puerperium and that the prevalence rate might be higher if a longer follow up period was adopted. In the present study, the PND prevalence of 15.7% at 2 months after delivery was more comparable with epidemiological studies of the non-Chinese populations that 10 to 15% of recently delivered women were affected by postnatal depression [27-29]. Postnatal depression can impose considerable family vulnerability and distress as well as tension within a marriage [8,30]. In its severe form, PND can eventually end up with suicide and infanticide. Because of its high prevalence, potential adverse consequences, and treatment availability, the early identification and management of PND is of imminent importance for the health of pregnant women and their families. Researchers investigating postnatal depression in the Chinese populations opined that Chinese women tend to keep their feelings and might choose to keep silent instead of taking the initiative to admit that they have problems so their depressive symptoms are under-reported [3,31]. Therefore, the active screening and identification of PND by healthcare professionals are particularly essential for the Chinese populations.

There are screening programmes for depression of women during the postnatal period in different places. In Hong Kong, the implementation of the CCDS since 2005 aims to serve the purpose of identifying PND early at MCHCs, where postnatal Chinese women are screened for postnatal depression with the Chinese version of the EPDS [15-19]. Those screened positive are assessed by psychiatric nurses at MCHCs and those deemed in need of further psychiatric management are referred to psychiatrists. To identify postnatal depression early, apart from implementing a screening programme with the use of EPDS for postnatal women, it is important to determine risk factors for postnatal depression so that Chinese women with these risk factors are monitored more closely [4,32].

Studies for exploring the antenatal risk factors for postnatal depression in the Chinese populations are relatively scarce, not up to date, and without using standardized diagnostic schedules and the most recent large scale one was by Lee et al. 2004 [9,14]. With the implementation of the CCDS for the early identification and management of PND in primary healthcare settings since 2005, it is the time to re-assess the risk factors for postnatal depression for Chinese women at the community level (i.e. at MCHCs where a significant proportion of women receive antenatal care). The results of this study serve as the blueprint for the further modifications of the service delivery model of perinatal psychiatric services for Chinese women.

### Risk factors for postnatal depression

In the present study, marital dissatisfaction (Relative Risk = 8.27), dissatisfied relationship with mother-in-law (Relative Risk = 3.93), antenatal depressive symptomatology (Relative Risk = 3.90), and anxiety-prone personality (Relative Risk = 2.14) predicted PND independently. Marital dissatisfaction has been consistently found to be an important determinant of postnatal depression [9,11,12]. Dissatisfied relationship with mother-in-law is a salient risk factor for postnatal depression in the Chinese populations [9,14]. In the traditional Chinese culture, after marriage, a woman would be named under her husband's surname and move to live with her husband's family. Moreover, the woman would become the "subordinate daughter" of her mother-in-law and be responsible to take care of her husband's family members. Under the role of a "subordinate daughter", the woman is supposed to respect and obey the opinions and advices from her mother-in-law in different aspects of her life including the ways to take care of the family and the newborn child [9,14,31]. Overt and covert conflicts would then emerge if the woman has different views with her mother-in-law and these conflicts can impose significant distress in the woman. Despite that Hong Kong has been developed as a modern city in the recent decades and that many women in Hong Kong now would not live with her mother-in-law after marriage, the traditional Chinese beliefs that a woman would be "subordinate" to her mother-in-law still prevail in some Chinese families. After the birth of a child, a woman would have increased contact with her mother-in-law if her mother-in-law provides peiyue support or if her mother-in-law pays visits to the newborn child at the woman's home. Because of the increased contact, conflicts on the ways of childcare between the woman and her mother-in-law become prominent and the adverse effects of these conflicts exacerbate if the woman could not gain the support from her husband. It is therefore not surprising that dissatisfied marital relationship and dissatisfied relationship with mother-in-law contribute significantly for the development of PND in Chinese women.

In the study by Lee et al. (2004) on Chinese women, conflicts with mother-in-law, marital dissatisfaction, past depression and antenatal depressive symptomatology were found to be independent antenatal risk factors for postnatal depression [9]. The results of the present study replicated some of the findings of Lee et al. in that marital dissatisfaction, dissatisfied relationship with mother-in-law and antenatal depressive symptomatology independently predicted PND [9,14]. Though past history of depression was associated with an increased risk for PND by univariate analysis, it was not an independent risk factor for PND in multivariate analysis in the present study. This negative finding might be due to the relatively small proportion (8.1%) of women suffered from a past history of depression in the present study. Instead, anxiety-prone personality was found to have predicted PND independently in the present study in which 62.7% of women with postnatal depression had anxiety-prone personality. The result echo with studies in the non-perinatal contexts that anxiety-prone personality is associated with depression [33-35]. The findings of the present study point to the importance of assessing antenatal depressive symptomatology and close monitoring of Chinese women with significant antenatal depressive symptomatology (e.g. those with antenatal EPDS score > 9) during the postnatal period for PND. Chinese women with anxiety-prone personality and relationship problems with husband and or mother-in-law also warrant close follow up. Moreover, antenatal interventions aiming at reducing these risk factors may serve to lower the chance of developing PND.

In Lee et al. study (2000), spouse dissatisfaction with the female gender of the baby increased the risk for PND [12]. In the traditional Chinese culture, a boy was more treasured than a girl as a boy signifies the promulgation of the family to the next and subsequent generations. However, the present study did not show that spouse dissatisfaction with the gender of the baby was a significant risk factor for PND. On the other hand, we found that quite a large number of participants and their spouse preferred to have a girl rather than a boy despite that they were not dissatisfied with the male gender of the baby. They opined that a daughter might be more obedient and attached to the family and that a daughter might be more caring for her parents when they get old. This phenomenon may reflect a change of values in the Chinese society in recent years that instead of the gender, the character and relationship of the child with the family are considered by parents as the most preferred attributes of the child. It would be important to evaluate in future studies whether this change in cultural preference of offspring gender may also exist in other Chinese communities, especially in the Mainland China with the implementation of its one-child policy.

The results of the present study are in consonance with the results of Lee et al. (2004) in that the presence of peiyue lowered the risk for PND but did not contribute to predict PND independently in multivariate analysis [9]. Peiyue is a Chinese postpartum custom of mandated family support during which recently delivered women are regarded as vulnerable and are exempted from their usual household duties in the month after delivery with the continual presence of a designated elder female kin, most commonly the women's mother or mother-in-law [9]. Lee et al. (2004) pointed out that if the mother-in-law was the peiyue support and the new mother did not get along well with her mother-in-law, the in-law tension could offset the potential benefit of the peiyue arrangement. In this study, the majority of women had peiyue support by their mother (39.6%) followed by their mother-in-law (25.8%) and 4.2% had support by peiyue maid. Some participants indicated that during the peiyue period they felt tired in dealing with their relationship problems and conflicts with their mother-in-law over child care and these conflicts made the women become distressed. On the other hand, in the present study, about half of those having peiyue maids as the peiyue support mentioned that the peiyue maids were not very helpful and skilful and that being with a person that they knew little before delivery made them feel uneasy. Peiyue maid is a relatively new occupation that has emerged in the recent years in the Chinese populations for the support of women during the peiyue period. The peiyue maid is trained to take care of the new mother, cook for her and assist her in child care. Therefore, we believe that the quality of peiyue support was much more important than the presence or absence of peiyue support. Moreover, the training programmes for peiyue maids may need to be enhanced.

Persistent wound (episiotomy wound or Caesarean section wound) pain for more than 2 weeks was found to be a significant risk factor in univariate analysis in this study despite that it did not predict PND independently. The relationships between pain and depression have been discussed extensively in the literature [36-38]. As compared with people without depression, people with depression reported more pain symptoms and depression and pain may share common pathogenic pathways with the involvement of serotonin. Moreover, the presence of pain may predict worse treatment response for depression [38]. Therefore, postnatal Chinese women with the presence of persistent episiotomy or Caesarean section wound pain need adequate wound care and pain control.

### Limitations

The generalizability of the results of this study was limited by the non-random nature of the sample recruited. Moreover, postnatal depression was assessed at around 2 months after delivery and those with onset of depression later than 2 months after delivery were not detected in this study. Two months after delivery was chosen as the time for assessment of PND in this study because it usually occurs in the first 2 months after delivery and Chinese women in Hong Kong usually go back to work 2 to 2.5 months after delivery and it is difficult to arrange an interview with them after they return to work. The strength of this study was that it was conducted in the community and a structured diagnostic interview was used to confirm the diagnosis of PND. A future study with a longer postnatal follow up period is warranted to further explore the risk factors for PND in the Chinese populations.

### Clinical implications

The findings in the present study support the importance of assessing antenatal depressive symptomatology and monitoring Chinese women with significant antenatal depressive symptoms (e.g. those with antenatal EPDS score > 9) closely during the antenatal and postnatal period for depression. In addition, healthcare professionals need to pay particular attention and closely follow Chinese women with anxiety-prone personality, marital dissatisfaction, and dissatisfied relationship with mother-in-law during the antenatal and postnatal period in order to detect PND early.

The results of this study serve as a basis for the further modifications of the service delivery model of the perinatal psychiatric services for Chinese women in Hong Kong. Apart from the screening of PND in primary healthcare settings in the community, the assessments of independent risk factors for PND should also be performed on pregnant Chinese women. With a closer and more frequent monitoring of pregnant women with the independent risk factors found in this study, PND might be identified and managed even earlier. Moreover, the incorporation of programmes and interventions aiming to reduce the impact of these risk factors on the women and their family may help to minimize the chance of the subsequent development of PND.

## Conclusions

Chinese women tend to keep their own feelings and emotions and it is important to monitor Chinese pregnant women with these predictive risk factors so that PND can be identified early.

## Competing interests

The authors declare that they have no competing interests.

## Authors' contributions

BWMS made the study designs, conceptualized the report, did the data analyses, data interpretation, and wrote the report. SSLL, PI, SFH, MWO made the study designs and critical appraisals of the report. All authors read and approved the final manuscript.

## Pre-publication history

The pre-publication history for this paper can be accessed here:

http://www.biomedcentral.com/1471-244X/12/22/prepub
